# Comparing the effectiveness of capecitabine versus 5-fluorouracil/leucovorin therapy for elderly Taiwanese stage III colorectal cancer patients based on quality-of-life measures (QLQ-C30 and QLQ-CR38) and a new cost assessment tool

**DOI:** 10.1186/s12955-015-0261-1

**Published:** 2015-05-19

**Authors:** Jen-Kou Lin, Elise Chia-Hui Tan, Ming-Chin Yang

**Affiliations:** Faculty of Medicine, School of Medicine, National Yang-Ming University, Taipei, Taiwan; Section of Colon and Rectal Surgery, Taipei Veterans General Hospital, Taipei, Taiwan; Institute of Health Policy and Management, College of Public Health, National Taiwan University, Taipei, Taiwan

**Keywords:** Quality of life, Colorectal cancer, Elderly, Treatment cost

## Abstract

**Background:**

Colorectal cancer (CRC) is a leading cause of cancer-related deaths in developed countries and its incidence increases with age. Intravenous administration of bolus 5-fluorouracil (5-FU) and leucovorin (LV) has been a standard treatment regime for stage III CRC. However, patients generally prefer oral therapy such as Capecitabine. Studies showed that combination of oxaliplatin and capecitabine demonstrated efficacy and safety on par with treatment involving various 5-FU/LV-based regimens in elderly patients as they are in younger ones. However, little is known regarding the cost of adjuvant therapy or the effect of therapy on HRQoL. Thus the aims of this study were to evaluate the influence of different adjuvant care for stage III CRC on the HRQoL of elderly patients and to compare the economic costs associated with capecitabine-based and 5-FU/LV-based adjuvant treatments from a societal perspective in Taiwan.

**Methods:**

A prospective, open-label, observational, multicenter study involving 123 patients aged 70 and over from 11 different centers was conducted between July 2008 and July 2011 in Taiwan. The adjusted monthly costs per patient and HRQoL were evaluated from individual-level data. The HRQoL of patients was assessed before and after adjuvant treatment. Direct and indirect costs of adjuvant treatment were estimated from a number of sources, and QoL scores were compared between groups.

**Results:**

After correcting for baseline characteristics of patients, no significant differences were observed in the global HRQoL scores between treatment groups during the study period. According to QLQ-CR38 results, capecitabine-based therapy appeared to alleviate problems related to defecation (4.54 *vs.* 8.5; *P* = 0.011); however, micturition problems increased (9.27 *vs.* 7.51; *P* = 0.04), compared with 5-FU/LV-based treatment. The adjusted monthly treatment cost per patient was NT$27,300 for capecitabine-based treatment and NT$53,671 for 5-FU/LV-based treatment. The total cost of 5-FU/LV-based treatment was 59 % greater than that of capecitabine-based treatment.

**Conclusions:**

Analyzing from the societal perspective in Taiwan, capecitabine-based therapy incurred lower treatment costs than 5-FU/LV-based therapy and did not jeopardize HRQoL. Therefore, capecitabine, with or without oxaliplatin, could be considered as an alternative treatment option for elderly patients with stage III CRC.

## Background

Colorectal cancer (CRC) is a leading cause of cancer-related deaths in developed countries and its incidence increases with age. Every year, approximately 1 million patients are diagnosed with CRC and half a million deaths are attributed to this disease worldwide [1]. In Taiwan, CRC is the second most frequently diagnosed cancer and the third leading cause of cancer-related death, after lung and liver cancer. The incidence of CRC in Taiwan has been increasing over the last 2 decades, with 11,004 cases recorded in 2008 [2]. Age-standardized mortality for CRC was 44 per 100,000 for men and 32 per 100,000 for women. Nearly 45 % of CRC patients are aged above 70 years, and the median age at death from advanced CRC is 73 years [3]. In the United States (US), national expenditures related to CRC care amounted to 12.16 billion dollars and lost productivity owing to deaths caused by CRC amounted to 10.65 billion dollars in 2010 [4]. With an aging population, CRC is expected to become increasingly common among older individuals, and subsequently increase the burden on health care resources.

Surgery remains the most common treatment for CRC; 53.1 % of all CRC patients in the US have undergone surgery [5]. Adjuvant chemotherapy after tumor resection is now considered a standard treatment for stage III CRC to prevent recurrence and prolong survival [6]. Compared to surgery alone, adjuvant chemotherapy or radiotherapy improves overall survival and reduces the probability of CRC recurrence. However, chemotherapy has been provided less frequently to elderly patients than those in other age groups [7].

Intravenous administration of bolus 5-fluorouracil (5-FU) and leucovorin (LV), either weekly or monthly over a period of 6 to 8 months, has been a standard treatment regime for stage III CRC [8]. The results from landmark trials have shown a 22 % reduction in mortality due to 5-FU/LV treatment versus surgery alone [9]. Adjuvant 5-FU/LV reduces the risk of relapse and prolongs patient survival after surgery [10]. Although the clinical benefits of adjuvant 5-FU/LV-based treatment are significant, data suggest that patients generally prefer oral therapy to intravenously administered treatments [11].

Capecitabine is an oral fluoropyrimidine designed to deliver 5-FU to tumor tissue. Many studies have shown that, when used an adjuvant treatment for stage III CRC, capecitabine therapy is at least as effective and well tolerated as 5-FU/LV alone [12]. These trials further determined that capecitabine provided equivalent disease-free survival (DFS) and overall survival (OS) rates among patients aged ≥70 years [13]. In a series of studies on stage II/III CRC, combination oxaliplatin and capecitabine chemotherapy demonstrated efficacy and safety on par with treatment involving various 5-FU/LV-based regimens [14].

To gain a better understanding of how cancer, surgery, and adjuvant therapy influence patient outcomes, objective clinical endpoints, including survival, postoperative disability, or death are increasingly being augmented by data associated with health-related quality-of-life (HRQoL). The American Society of Clinical Oncology considers that patient outcomes such as toxicity, survival, and HRQoL are more important than cancer outcomes. Thus, HRQoL assessment is essential to the clinical decision-making process as it provides insights regarding patient experience with disease and treatment [15]. Commella and colleagues compared oral capecitabine plus oxaliplatin and 5-FU/LV plus oxaliplatin treatments for MCRC patients [16], and found that there were no differences in the QoL of patients treated using these regimens. Venderbosch et al. evaluated the effect of first-line capecitabine monotherapy and other capecitabine combination treatments in different age groups with MCRC [17]. The results showed that among 3 patient age groups (>75, 70–75 and <70 years), there were no differences in global QoL. Hus and colleagues estimated the cost-effectiveness of oral capecitabine in the adjuvant treatment of stage III colon cancer patients in Taiwan [18]. With regard to the perspectives of the National Health Insurance Administration (NHIA) and society in Taiwan, they found that capecitabine treatment was less expensive than 5-FU/LV, and the overall direct cost with capecitabine treatment was less than that with 5-FU/LV (NT$129,327 vs. NT$233,873) during the 24-week treatment period. Lang and colleagues estimated that the undiscounted, average 10-year cancer-related medical care cost for CRC was NT$584,985 in 2002 [19].

Previous studies have demonstrated that the clinical benefits from adjuvant treatments are at least as significant in elderly patients as they are in younger ones. However, little is known regarding the cost of adjuvant therapy or the effect of therapy on HRQoL. Thus the aims of this study were to evaluate the influence of different adjuvant care for stage III CRC on the HRQoL of elderly patients and to compare the economic costs associated with capecitabine-based and 5-FU/LV-based adjuvant treatments from a societal perspective in Taiwan.

## Methods

### Ethical standards

The study was approved by the ethics board of each institution: Taipei Veterans General Hospital (approval numbers: 98-06-26A), Shin Kong Wu Ho-Su Memorial Hospital (approval numbers: 97E-018), Cathay General Hospital (approval numbers: CT9738), Mackay Memorial Hospital (approval numbers: MM-I-S-607H), Chia-Yi Christian Hospital (approval numbers: 97040), Changhua Christian Hospital (approval numbers: CCH081009), National Cheng Kung University Hospital (approval numbers: ER-97-116), China Medical University Hospital (approval numbers: DMR97-IRB-156), Taipei Municipal Wanfang Hospital (approval numbers: 97043), Chiayi Chang Gung Memorial Hospital and Kaohsiung Chang Gung Memorial Hospital (approval numbers: 972003B [for two hospitals with a common ethics board]). All the participants gave written informed consent prior to the commencement of the study.

### Patient population

This study included 11 institutions: 9 academic medical centers and 2 regional hospitals. Patients aged 70 and over with stage III CRC, as confirmed by physicians or by a pathological examination in these institutions, and who underwent potentially curative resection of the tumor were eligible for the study. In addition, patients had to be prescribed capecitabine or 5-FU/LV, either with or without or oxaliplatin.

Patients who had a history of other malignancies during the study period were excluded from this study. Other exclusion criteria were participation in any investigational drug study 4 weeks before the start of the treatment, having received adjuvant radiotherapy in combination with chemotherapy after resection, or any changes in the use of study drugs during the period of study.

### Study design

This was a prospective, open-label, observational, multicenter study. The objectives were to evaluate changes in HRQoL and to determine the costs of various adjuvant treatments administered to stage III CRC patients. Demographic data and clinical characteristics as well as HRQoL and cost data were collected for analysis.

Two adjuvant chemotherapy regimens were administered and tested: capecitabine with or without oxaliplatin (case group) and 5-FU/LV with or without oxaliplatin (comparison group). We collected patients’ demographic characteristics, including age, gender, education level, marital status, work status and family history, when interviewing at baseline survey. Their clinical characteristics including comorbidity, used oxaliplatin or not and with stoma or not were collected by chart review. We recorded the baseline values for HRQoL before treatment as well as 30 days after treatment, rather than immediately after treatment, because HRQoL status remains in a state of transition owing to the ongoing effects of medication. Treatment was continued for 6 months or until disease remission, whichever occurred first.

Between July 2008 and December 2010, eligible patients were assigned to either the capecitabine or 5-FU/LV treatment group by their oncologist and followed up for 7 months, until the clinical cut off in July 2011. In Taiwan, the chemotherapy treatment could be administered either in an inpatient setting or using a chemotherapy pump with 72 h infusion depending on patient’s age, capable to take care chemo pump and distance from their home to the hospital. In order to capture the real utilization of CRC patient received adjuvant treatment, all utilization including frequency of outpatient visit and number of admissions, length of stay of each hospitalization and so on were collected. The total duration of the study was 25 months. Questionnaires were administrated by nurses, all of whom were actively involved in the study, at each hospital and filled out by each patient. Nurses provided assistance in filling out the questionnaires when necessary.

### HRQoL measures

The questionnaires frequently used to assess the HRQoL of CRC patients are the QoL QLQ-C30, developed by the European Organization for Research and the Treatment of Cancer (EORTC), the Chinese version of this document [20], the colorectal module QLQ-CR38 [21], and the Functional Assessment of Cancer Therapy-General version (FACT-G). According to Uwer et al., the EORTC QLQ-C30 is well suited for evaluating patients receiving chemotherapy and the colorectal module QLQ-CR38 provides additional clinically relevant information. Therefore, we selected the EORTC QLQ-C30 and QLQ-CR38 questionnaires to investigate the HRQoL of the CRC patients.

The former contains 30 questions, subdivided into 5 functional levels (physical, role, emotional, cognitive, and social), 9 symptom scales (fatigue, nausea and vomiting, pain, dyspnea, insomnia, appetite loss, constipation, diarrhea, and financial difficulties) as well as a single global QoL scale. Scores are summed and transformed to a value ranging from 0 to 100 based on the EORTC QLQ-C30 scoring manual [22]. A high score on the functional scale represents a high level of function in daily life, a high score on the global QoL scale represents a high QoL, and a high score on the symptom scale represents a high level of symptomatology or other health problems.

The EORTC QLQ-CR38 comprises 38 questions, of which 19 were completed by all study participants, while the remaining 19 questions were divided into groups relevant to various subsets of patients (e.g., male or female, the presence or absence of stoma). This questionnaire is subdivided into 4 functional scales (body image, sexual functioning, sexual enjoyment, and future perspective), 8 symptom scales (micturition problems, gastrointestinal tract symptoms, chemotherapy side effects, defecation problems, stoma-related problems, male and female sexual problems, and weight loss). The validity and reliability of this tool has been established in a study involving Dutch CRC patients. The scoring for this questionnaire involves the same methodology as that used for the EORTC QLQ-C30 questionnaire [22].

Questionnaire manuals dictate that missing values should be dealt with as follows: if at least half of the items on a scale are completed, the scale score is divided by the number of items present; if fewer than half of the items are completed, the scale is considered missing.

### Minimum important difference (MID)

MID is defined as the smallest difference in score for the domain of interest, which is considered important by patients (beneficial or harmful) and would lead a clinician to consider altering the course of treatment. With regard to the EORTC QLQ-C30, Osoba et al. suggested that an MID of more than 10 points between baseline and any subsequent visit could be considered a clinically significant change [23].

### Direct medical and non-medical costs

For this study, CRC patients (ICD-9-CM codes: 153, 154) were defined as those who received capecitabine-based or 5-FU/LV-based treatment after surgery. Patients who received post-surgical radiotherapy were excluded. Data related to use and expenditure related to medical and non-medical services were collected directly from self-administered questionnaires. Patients were asked to recall events related to treatment episodes. This study gathered the medical utilization records of patients, including outpatient visits related to chemotherapy, other outpatient visits, length of inpatient chemotherapy stay, emergency visits, and treatment for adverse events. The average expenditure related to each of these services was calculated from records obtained from the Longitudinal National Health Insurance Research Database (NHIRD) for 2007 and 2008. This database contains data on hospital admission and ambulatory visits, primary and community care services as well as prescribed medication for the 1 million individuals covered under the comprehensive reimbursement schemes of the National Health Insurance Administration in Taiwan. Finally, direct medical costs were obtained by multiplying the average medical expense by the number of instances of each type of medical service.

Direct non-medical utilization costs include the cost of transportation associated with receiving medical care, home nursing fees, and special supplements or food required during treatment. Direct non-medical costs were also determined from self-administered questionnaires. All the costs were adjusted for patient’s demographic and clinical characteristics listed in Table 1 by multivariate linear regression and were not discounted during study period.

**Table 1:** Characteristics of stage III CRC in elderly patients

Variable		Capecitabine group		5-FU/LV group		*P* value
		n	%	n	%	
n		93	75.61	30	24.39	
Age (mean, SD) ^a^		(77.47, 4.53)		(75.27, 4.35)		0.021
Gender ^b^						
	Male	46	49.46	24	80.00	0.005
	Female	47	50.54	6	20.00	
Education ^b^						
	No more than junior high school	83	90.22	18	62.07	0.002
	Senior high school	6	6.52	7	24.14	
	University or above	3	3.26	4	13.79	
Marital status ^b^						
	Married or cohabiting	70	81.40	25	83.33	1.000
	Other	16	18.60	5	16.67	
Employment status ^b^						
	Unemployed	90	96.77	26	89.66	0.1453
	Employed	3	3.23	3	10.34	
Tumor site ^b^						
	Colon	77	82.80	19	63.33	0.784
	Rectum	16	17.20	11	36.67	
Family history of cancer ^b^						
	No	75	82.42	23	79.31	0.040
	Yes	16	17.58	6	20.69	
Comorbidity ^b^						
	None	27	29.67	10	33.33	0.820
	Hypertension	37	40.66	10	33.33	0.524
	CAD	8	8.79	3	10.00	1.000
	CVA	9	9.89	-	-	0.110
	Renal insufficiency	6	6.59	2	6.67	1.000
	DM	18	19.78	6	20.00	1.000
	COPD	3	3.30	1	3.33	1.000
	Liver cirrhosis	5	5.49	-	-	0.331
	Other comorbidity	27	29.67	8	26.67	0.820
Oxaliplatin administered ^b^						
	No	82	88.17	10	33.33	< 0.0001
	Yes	11	11.83	20	66.67	

Abbreviations: *CAD* coronary artery disease, *CVA* cardiovascular disease, *DM* diabetes mellitus, *COPD* chronic obstructive pulmonary disease

^a^ Student *t*-test was used to calculate the statistical significance at the 5 % level

^b^ Pearson chi-square test was used to calculated the significance at the 5 % level

### Societal costs

We estimated the cost of lost patient productivity associated with receiving outpatient or inpatient care. We also estimated the cost of lost productivity for relatives who accompanied patients to treatment events. The followings are the formulas of calculating: (1) loss of productivity incurred by patients who received outpatient visit or inpatient care; and (2) loss of productivity of accompanying relatives:

1. Loss of productivity of patients receiving outpatient or inpatient care = (Average income * Labor force participation rate*(1- Unemployment rate)/Average work hours (181.2 h))*Time spent for medical treatment
2. Loss of productivity of accompanying relatives = (The minimum wage in 2010 (NT$ 17280)/Average work hours (181.2 h))*Time spent for accompanying

These data was retrieved from the self-administered questionnaire and expected losses were calculated as income loss. In accordance with the human capital approach, expected losses in productive time were translated into monetary terms. The age- and sex-specific average income, labor force participation rates, and unemployment rates were obtained from the 2010 Report on the Manpower Utilization Survey to estimate lost productivity for accompanying relatives [24].

### Statistical methods

Descriptive analysis was used to compare baseline characteristics between groups undergoing capecitabine- and 5-FU/LV-based treatments. Categorical variables were summarized in frequency tables, and continuous and other numeric variables were summarized by presenting the number of observations, the mean value, and the standard deviation. Student *t*-test and Pearson chi-square test were used to examine the statistical significance of baseline characteristics between these two groups. Paired *t*-test was used to examine the HRQoL scores between baseline and the 28th week of these two treatments. A mixed model adjusting for patient demographic and clinical factors was assembled to test the change in HRQoL from baseline to 28th week between these two groups.

## Results

A total of 123 elderly patients (capecitabine group, n = 93; 5-FU/LV group, n = 30) completed all 3 surveys. The characteristics of these patients at baseline are presented in Table 1. After adjustment, 108 of the patients were included in the analyses (81 from the capecitabine group and 27 from the 5-FU/LV group). There was a significant difference with respect to age, gender, education, family history, and use of oxaliplatin between patients receiving capecitabine- and 5-FU/LV-based treatments. Compared with the 5-FU/LV group, patients in the capecitabine group were older (77.47 *vs.* 75.27 years; *P* = 0.021), had a higher percentage of female patients (50.54 % *vs.* 20.00 %; *P* = 0.005) and junior high school graduates (90.22 % *vs.* 62.07; *P* = 0.002), had a lower frequency of family history of cancer (82.42 % *vs.* 79.31 %; *P* = 0.04), and were less frequently administered oxaliplatin (88.17 % *vs.* 33.33 %; *P* < 0.0001).

### General HRQoL

A comparison of the EORTC QLQ-C30 results between the capecitabine and 5-FU/LV groups is presented in Table 2. After corrections for age, gender, education, marital status, employment status, tumor site, family history, comorbidities, and use of oxaliplatin during adjuvant treatment, no significant differences were observed between the 2 groups at baseline. Significant improvements in physical function, role function, emotional function, global health status, and fatigue were reported by both groups between baseline and the final visit. Compared with the 5-FU/LV-based treatment group, patients in the capecitabine-based treatment group showed significant improvements in cognitive function, pain, dyspnea, and insomnia, but significantly reduced social function between baseline and the final visit. We also analyzed the adjusted means in the final visit after correction for patient characteristics and QLQ - C30 scores at baseline, after which no significant differences were observed between the results of the 2 groups.

**Table 2:** Results for health-related quality of life from the QLQ-C30

Dimensions		Capecitabine-based				5-FU/LV-based				*P* value^b^
		n	Mean	SD	*P* value^a^	n	Mean	SD	*P* value^a^	
Physical										
	Baseline	81	73.90	11.78		27	80.12	12.46		0.746
	28th week	81	86.14	11.13	<0.0001	27	86.12	11.48	0.038	0.657
Role										
	Baseline	81	78.58	11.65		27	77.48	14.78		0.865
	28th week	81	90.97	11.08	<0.0001	27	86.77	14.31	0.001	0.582
Emotional										
	Baseline	81	84.45	8.54		27	83.88	7.57		0.537
	28th week	81	90.24	7.56	<0.0001	27	89.93	6.10	<0.0001	0.357
Cognitive										
	Baseline	81	88.18	7.42		27	88.58	8.30		0.091
	28th week	81	92.55	7.29	<0.0001	27	87.77	6.93	0.641	0.331
Social										
	Baseline	81	83.15	8.58		27	82.75	11.85		0.313
	28th week	81	84.86	9.06	0.226	27	91.03	8.98	0.002	0.532
Global health status										
	Baseline	81	62.93	6.59		27	59.61	8.98		0.517
	28th week	81	75.58	5.64	<0.0001	27	74.76	4.97	<0.0001	0.854
Fatigue										
	Baseline	81	25.11	10.49		27	25.80	8.81		0.884
	28th week	81	9.34	10.53	<0.0001	27	17.59	9.63	0.002	0.259
Nausea and Vomiting										
	Baseline	81	3.91	9.41		27	6.77	10.74		0.467
	28th week	81	6.56	9.96	0.002	27	6.81	10.89	0.982	0.187
Pain										
	Baseline	81	11.75	8.36		27	8.41	7.61		0.421
	28th week	81	4.63	6.24	<0.0001	27	4.07	6.13	0.058	0.746
Dyspnea										
	Baseline	81	8.82	8.62		27	6.00	9.61		0.087
	28th week	81	6.64	5.13	0.029	27	4.90	8.52	0.536	0.689
Insomnia										
	Baseline	81	22.16	15.78		27	18.50	13.71		0.050
	28th week	81	16.15	11.96	0.002	27	17.19	11.73	0.603	0.382
Appetite loss										
	Baseline	81	10.44	10.91		27	19.64	12.17		0.004
	28th week	81	11.10	14.75	0.696	27	11.67	16.08	0.006	0.476
Constipation										
	Baseline	81	14.31	12.40		27	6.36	11.67		0.057
	28th week	81	14.28	7.64	0.982	27	6.24	6.53	0.951	0.273
Diarrhea										
	Baseline	81	13.88	16.39		27	13.82	14.39		0.581
	28th week	81	9.05	14.78	0.002	27	12.91	13.16	0.783	0.276
Financial difficulties										
	Baseline	81	6.06	6.70		27	7.45	7.24		0.666
	28th week	81	6.05	10.20	0.990	27	4.83	10.33	0.118	0.040

^a^ Paired *t*-test was used to calculate the statistical significance at the 5 % level between the adjusted means at baseline and the 28th week

^b^ Mixed model was used to calculate the statistical significance at the 5 % level following adjustment for patient age, gender, education, marital status, work status, tumor site, family history, comorbidities, use of oxaliplatin, and baseline HRQoL score

### Cancer-specific HRQoL

A comparison of the EORTC QLQ - CR38 results between the capecitabine- and 5-FU/LV-based treatment groups is shown in Table 3. Patients in the capecitabine-based group suffered fewer side effects of chemotherapy (6.92 *vs.* 16.79; *P* = 0.003) and fewer problems related to defecation (5.38 *vs.* 9.05; *P* = 0.025) at baseline. After correction for patient characteristics and baseline HRQoL scores, patients in the capecitabine group still suffered fewer problems with defecation (4.54 *vs.* 8.5; *P* = 0.011); however, they experienced more micturition-related difficulties (9.27 *vs.* 7.51; *P* = 0.04) than the 5-FU/LV group in the final visit.

**Table 3:** Results for health-related quality of life from the QLQ-CR38 questionnaire

		Capecitabine-based				5-FU/LV based				*P* value^b^
		n	Mean	SD	*P* value^a^	n	Mean	SD	*P* value^a^	
Body image										
	Baseline	81	97.38	6.14		27	92.53	7.50		0.174
	28th week	81	98.04	8.25	0.274	27	94.05	7.68	0.120	0.257
Future perspective										
	Baseline	81	54.52	15.93		27	66.70	18.30		0.135
	28th week	81	67.66	13.61	<0.0001	27	67.70	15.49	0.769	0.422
Sexual functioning										
	Baseline	81	83.15	8.58		27	82.75	11.85		0.313
	28th week	81	84.86	9.06	0.226	27	91.03	8.98	0.002	0.159
Micturition										
	Baseline	81	14.23	8.44		27	13.50	7.46		0.891
	28th week	81	9.27	6.88	<0.0001	27	7.51	6.33	0.000	0.040
Chemotherapy side effects										
	Baseline	81	6.92	7.03		27	16.79	8.37		0.003
	28th week	81	7.50	7.38	0.635	27	11.50	8.03	0.008	0.706
Gastrointestinal tract										
	Baseline	81	9.27	7.79		27	12.24	7.55		0.277
	28th week	81	4.42	6.90	<0.0001	27	6.09	6.56	<0.0001	0.163
Defecation problems										
	Baseline	81	5.38	11.46		27	9.05	9.60		0.025
	28th week	81	4.54	7.14	0.205	27	8.50	5.52	0.683	0.011
Weight loss										
	Baseline	81	16.41	13.31		27	24.86	14.73		0.973
	28th week	81	6.42	12.41	<0.0001	27	7.47	8.52	<0.0001	0.309

^a^ Paired *t*-test was used to calculate the statistical significance at the 5 % level between the adjusted means at baseline and the 28th week

^b^ Mixed model was used to calculate the statistical significance at the 5 % level after adjustment for patient age, gender, education, marital status, work status, tumor site, family history, comorbidities, use of oxaliplatin, and baseline HRQoL score

### Societal prospective-direct and indirect costs

The monthly societal costs for the capecitabine and 5-FU/LV groups during adjuvant chemotherapy are summarized in Table 4. The total cost per patient in the 5-FU/LV group was higher than that in the capecitabine group, with respect to both direct costs (medical and non-medical expenditures) and indirect costs (loss of productivity for the patient and accompanying relatives). After correction for patient characteristics, the per-patient costs in the capecitabine group amounted to NT$17,200.46 (direct) and NT$27,299.60 (indirect), totaling to NT$42,372.52 per patient in monthly societal costs. The per-patient costs in the 5-FU/LV group were NT$42,372.52 (direct) and NT$11,298.55 (indirect), totaling to NT$53,671.07 per patient in monthly societal costs. Differences in expenditures associated with inpatient chemotherapy and side effects had the greatest influence on incremental costs in the 5-FU/LV arm.

**Table 4:** Monthly direct and indirect per patient costs of adjuvant treatment for stage III CRC

	Capecitabine-based group	5-FU/LV-based group	Unadjusted difference*	*P* value	Adjusted difference*	*P* value
	Mean (SD)	Mean (SD)				
Direct cost						
*Medical cost*						
Ambulatory cost	6410.32 (4116.36)	4636.80 (5041.69)	−1773.52	0.0548	−603.50	0.2981
Outpatient chemotherapy cost	2856.64 (6854.69)	10626.70 (17649.99)	7770.06	0.0249	1994.60	0.2555
Inpatient chemotherapy cost	462.59 (3200.74)	26386.21 (24329.36)	25923.62	<0.0001	14718.53	0.0009
Side effects of treatment cost	151.49 (1027.40)	11857.91 (10068.21)	11706.42	<0.0001	7324.83	<0.0001
*Non-medical cost*						
Travel cost	51.56 (64.34)	56.67 (138.52)	5.11	0.8464	2.52	0.8906
Nursing aide	397.85 (2863.34)	1366.67 (4802.90)	968.82	0.3023	723.86	0.0121
Alternative care	56.99 (333.10)	-	−56.99	0.1024	−46.21	0.0086
Total direct cost per patient	10387.44 (9016.48)	54930.95 (27747.10)	44543.51	0.3374	25172.06	<0.0001
Indirect cost						
Loss of productivity	862.01 (1250.5)	1654.77 (1168.69)	792.76	0.003	140.27	0.411
Loss of productivity for accompanying relatives	6502.90 (9445.88)	12474.00 (8802.9)	5971.10	0.003	1059.14	0.411
Total indirect cost per patient	7364.91 (10696.38)	14128.77 (9971.59)	6763.86	0.003	1199.41	0.411
Total cost per patient	17752.35 (12907.36)	69059.72 (26313.49)	51307.37	<0.0001	26371.47	<0.0001

* Values greater than zero reflect a higher monthly expenditure during 5-FU/LV regimens

## Discussion

The benefits of adjuvant treatment include reduced risk of relapse and prolonged survival but the side effects and complications often reduce the QoL for the patients. Although many studies have employed HRQoL measurements when studying CRC patients, however, only few studies addressed the impact of adjuvant treatments on HRQoL and the associated costs of caring for the elderly patients. To the best of our knowledge, this is the first study to examine both the QoL and societal cost with regard to the influence of adjuvant treatment on stage III colon and rectal cancer for patients aged ≥70 years. Results from the EORTC QLQ-C30 and CR-38 questionnaires demonstrated that capecitabine-based therapy was not inferior to 5-FU/LV-based therapy in terms of QoL. Moreover, patients undergoing capecitabine-based therapy incurred low treatment costs and showed improved conserved productivity. The QLQ-C30 questionnaire showed no difference in the QoL between groups, while the QLQ-CR38 indicated that the capecitabine group experienced problems associated with defecation and micturition after treatment.

Our results are in agreement with a randomized factorial trial involving elderly and frail MCRC patients conducted in the UK which showed no differences in the QoL between patients receiving capecitabine (with or without oxaliplatin) and those receiving fluoropyrimidines (with or without oxaliplatin) [25]. A randomized phase III study, conducted in France, produced similar results, i.e., no significant difference in QLQ-C30 scores between patients undergoing XELOX or FOLFOX-6 treatment [26]. In the current study, after adjuvant therapy, both groups showed significant improvement in global health status, and no clinically relevant difference was found between these groups in the sub-categories in either the QLQ-C30 or QLQ-CR38 questionnaires. These results are not surprising, considering that capecitabine-based treatment (with or without oxaliplatin) and 5-FU/LV-based treatment (with or with oxaliplatin) have similar safety and efficacy profiles with regard to the elderly [27]. Finally, baseline QLQ-C30 and QLQ-CR38 scores were similar in the capecitabine and 5-FU/LV arms, except for the loss of appetite, side effects of chemotherapy, and problems with defecation. Patients in the capecitabine group experienced far less problems from these conditions than those in the 5-FU/LV group. However, with a difference in scores less than the MID of 10 points, the discrepancies are not clinically relevant.

Many studies have reported that capecitabine-based treatment is less expensive than other treatment regimens. One study conducted in the US showed that non-Medicare CRC patients treated with capecitabine had lower monthly total direct medical (US$740) and chemotherapy-related expenses (US$785) than patients treated with 5-FU/LV [28]. Another cost comparison reported that the monthly expenditure per patient for capecitabine (US$6,683) or capecitabine/oxaliplatin (US$11,436) treatment was significantly lower than that for 5-FU/LV (US$9,304) or 5-FU/LV/oxaliplatin (US$14,320) treatment [29]. A cost-benefit analysis conducted in the Netherlands showed that the monthly adjuvant treatment costs were €3,770 for capecitabine and €4,704 for 5-FU/LV [30]. In the UK, a cost-minimization study reported that the treatment costs for oral capecitabine (£2,132) were lower than those for the Mayo regimen (£3,593) over the same treatment period [31]. However, few studies provide a reasonable comparison between oral and intravenous chemotherapy costs for elderly CRC patients. In line with the findings of these studies, our results suggest that the use of capecitabine-based adjuvant chemotherapy after tumor resection in elderly patients with stage III colon cancer reduces societal costs, as compared to 5-FU/LV-based therapy. The adjusted monthly cost saving per patient is NT$26,372 (US$879) when a capecitabine-based treatment is used, mainly because of the significantly lower costs of inpatient chemotherapy and AE-related expenditure. In addition, our results show that patients treated with capecitabine-based therapy experienced a smaller loss of productivity that those treated with 5-FU/LV-based therapy.

When interpreting these results, several issues should be considered. First, the current study was an observational, non-randomized study. Although it reflects the actual use of societal resources for patient care, it is still subject to bias. The allocation of resources for specific treatment methods might have been biased by patient characteristics or socio-economic status. Therefore, we used a mixed model to adjust for potential bias. Second, very few patients responded to the sexual health-related dimensions of the QLQ-CR38 questionnaire. Therefore, the influence of treatment method on sexual enjoyment and sexual problems on the groups was not investigated. Third, the imbalance in these two groups might have impact on detecting a change of HRQoL scores. However, considering the MID of EORTC QLQ-C30, the estimate of power of this model was almost 0.99 which is enough to detect the change of HRQoL scores of patients between these two arms. Fourth, there is no suggested MID for the EORTC QLQ-CR38. We may identify the statistical difference, instead of clinical difference, between capecitabine-based therapy and 5/FU/LV-based therapy. Fifth, 66 % of the 5-FU/LV-based group combined with oxaliplatin therapy but only 11 % of the capecitabine-based group received oxaliplatin. The results of the study also indicated that the number of side effects in the capecitabine-based group was 0.02 ± 0.15 and was 1.68 ± 1.43 in the 5-FU/LV-based group. The occurrence of side effects may have potential influence on efficacy and toxicity, and consequently reflected on patient’s HRQoL and treatment costs.

## Conclusions

To the best of our knowledge, this is the first study to gather HRQoL and cost data from elderly patients at the individual level. Capecitabine-based therapy can be considered superior to 5-FU/LV-based therapy in terms of cost and at least as effective in terms of QoL when treating elderly patients with stage III CRC patients. Therefore, from both clinical and economic viewpoint, the capecitabine-based regimen, with or without oxaliplatin, could be considered as an appropriate alternative to the 5-FU/LV-based regimen.
